# Multi-Institutional Verification of a Novel Predictor (Volume-Scaled SUVmax) for Successful Biology-Guided Radiotherapy Delivery of Small Targets

**DOI:** 10.3390/cancers17223645

**Published:** 2025-11-13

**Authors:** M. Ramish Ashraf, Daniel Pham, Girish Bal, Huixiao Chen, Henry S. Park, Tyler Watkins, Bin Cai, Shahed N. Badiyan, Lucas K. Vitzthum, Billy W. Loo, Murat Surucu

**Affiliations:** 1 Department of Radiation Oncology, Stanford University School of Medicine, Stanford, CA 94305, USA; 2 RefleXion Medical, Hayward, CA 94545, USA; gbal@reflexion.com; 3 Department of Therapeutic Radiology, Yale University School of Medicine, New Haven, CT 06520, USA; huixiao.chen@yale.edu (H.C.); henry.park@yale.edu (H.S.P.); 4 Department of Radiation Oncology, City of Hope Medical Center, Duarte, CA 91010, USA; 5 Department of Radiation Oncology, University of Texas Southwestern Medical Center, Dallas, TX 75390, USA; bin.cai@utsouthwestern.edu (B.C.);

**Keywords:** biology-guided radiotherapy, PET, 3D printing, standard uptake value

## Abstract

Background/Objectives: The aim of this study was to establish the relationship between target size and the required diagnostic PET maximum standard uptake value (SUVmax) thresholds needed for successful Biology-guided Radiotherapy (BgRT) delivery on RefleXion X1 PET-linac. The current clinical eligibility recommendation is an SUVmax ≥ 6 at simulation, but the RefleXion system subsequently evaluates Activity Concentration (AC), which must exceed 5 kBq/mL for successful BgRT planning. Methods: A custom 3D-printed phantom containing six spherical targets (8 to 20 mm diameter) was used with varying target-to-background ratios (5:1 to 20:1) of 18F-FDG to systematically achieve a range of SUVmax values for each target size. Images were acquired on Siemens Biograph mCT for SUVmax quantification and RefleXion X1 for AC measurements. Twenty-four BgRT plans were evaluated, and delivery accuracy was validated using ArcCHECK. Additionally, retrospective data from 18 patients across four institutions were analyzed to validate the phantom-derived findings. Results: The PET-linac successfully planned treatments for 13/24 experiments, all achieving an AC > 5 kBq/mL. SUVmax requirements varied by target size: 16–20 mm targets required an SUVmax > 6, consistent with current recommendations, while smaller targets required higher thresholds (e.g., 13 mm: SUVmax > 10, and 11 mm: SUVmax > 15). 8 and 9 mm targets failed to meet AC requirements even at SUVmax 14. Successful deliveries maintained acceptable accuracy, with gamma passing rates of 92.4% ± 5.0% (3%/2 mm) and 97.6% ± 1.9% (3%/3 mm). Analysis revealed that Volume (cc) × SUVmax > 11 consistently predicted successful BgRT planning across all target sizes. This threshold was validated using multi-institutional PET-CT patient data (mean: 11.36, 95% CI: 9.1–12.9), correctly predicting treatment eligibility in 15 of 18 cases. Conclusions: Target size significantly influences BgRT eligibility. We derived a new criterion, Volume(cc) × SUVmax > 11 (95% CI: 9.1–12).

## Simple Summary

This study establishes size-dependent SUVmax thresholds for successful Biology-guided Radiotherapy (BgRT) delivery on RefleXion X1 PET-linac. While current clinical guidelines recommend an SUVmax ≥ 6, this threshold only applies to larger targets (16–20 mm). Using a 3D-printed phantom with six spherical targets (8–20 mm) and varying 18F-FDG concentrations, we demonstrated that smaller targets require significantly higher SUVmax values: 13 mm targets need an SUVmax > 10, and 11 mm targets require an SUVmax > 15. Targets ≤ 9 mm failed to achieve the required Activity Concentration (AC) > 5 kBq/mL even at SUVmax 14. We derived a new eligibility criterion, Volume(cc) × SUVmax > 11, which successfully predicted treatment feasibility across all target sizes. This threshold was validated using multi-institutional patient data from 18 cases, correctly predicting eligibility in 83% of patients. These findings provide crucial guidance for patient selection in BgRT, ensuring optimal treatment outcomes while avoiding futile planning attempts for inadequately sized or active targets.

## 1. Introduction

Biology-guided Radiotherapy (BgRT) represents a significant advancement in image-guided radiation therapy, where positron emission tomography (PET) signals from the tumor are used to guide treatment delivery in real time. The RefleXion X1 system (RefleXion Medical, Hayward, CA, USA) is the first clinical implementation of BgRT using SCINTIX technology [1,2,3,4,5]. The RefleXion X1 consists of a 6 MV-flattening filter-free (FFF) linear accelerator, dual 90° arcs of PET detectors, a binary multi-leaf collimator (MLC) capable of 100 Hz transitions, a 16-slice kilovoltage fan-beam CT scanner, and a megavoltage imaging panel. This integrated system rotates at 60 RPM and can deliver from 300 discrete firing positions around the patient. The treatment is delivered by stepping the couch to discrete locations for beam delivery, where the couch is moved in between delivery and not during delivery. The PET system provides a field of view of 50 cm in diameter using PET detectors with 5.2 cm in axial length, suitable for tumor sizes from 1 to 5 cm in the cranio-caudal direction [6].

The BgRT clinical workflow comprises several unique steps [7]. After conventional CT simulation and target contouring, a non-prescriptive structure called the Biology-Tracking Zone (BTZ) is created by adding a margin to the internal target volume (ITV). This volume focuses the BgRT delivery around the tumor and masks emissions from outside this region. Patients then undergo a separate “Functional Modeling” session on the Reflexion X1 system, capturing PET emissions essential for BgRT treatment planning. Unlike conventional radiotherapy planning, which optimizes fluence to achieve the desired dose distribution, BgRT backprojects PET images onto each firing angle and creates a “firing filter” that transforms these PET image projections into deliverable fluences. This process accommodates potential variations in PET target-to-background ratios, spatial uncertainties, and dose delivery fluctuations through bounded dose-volume histograms (bDVHs).

To assess whether a patient is a candidate for BgRT treatment, the current clinical guidance is that tumor size should be within 1 cm to 5 cm, there should be an SUVmax ≥ 6 at the time of diagnostic PET-CT, and the tumor should be >2 cm away from any PET-avid organs at risk [8]. Additionally, during the functional modeling session, Activity Concentration (AC) and Normalized Target Signal (NTS) are calculated to verify sufficient tumor visibility. AC quantifies the target’s detectability by measuring the signal-to-background contrast in kBq/mL, calculated as the difference between the mean activity in the top 80% of voxels within the Biological Tracking Zone (BTZ) and the mean activity in a surrounding background shell [9]. The BTZ typically encompasses the gross tumor volume (GTV or ITV) plus a 10 mm margin, while the planning target volume (PTV) is defined as GTV plus a 5 mm margin. For successful BgRT delivery, the system requires an AC > 5 kBq/mL. The normalized target signal (NTS) measures the signal-to-noise ratio which is calculated as the difference in the AC within the BTZ and the AC of the background shell divided by the standard deviation of the pixels in the background shell. An NTS > 2.7 is needed during functional modeling. Essentially, AC represents the contrast between target and background signals, while the NTS measures the relative contrast against background noise. On the day of each fraction, another PET scan is performed on Reflexion X1 (a pre-scan PET) to verify that the AC and NTS values are still within an acceptable range. An AC > 5 kBq/mL and NTS > 2.0 are needed during pre-scan at the time of treatment.

The SUVmax ≥ 6 criterion fails to account for target size or volume variations, which significantly impact PET signal detection, particularly for smaller lesions affected by partial volume effects. While larger tumors may readily achieve the SUVmax threshold, smaller targets may require substantially higher values to generate sufficient AC for successful BgRT tracked delivery. This size dependency creates a knowledge gap in patient selection, potentially excluding candidates with smaller lesions who might benefit from BgRT. This dependency may also lead to unsuccessful planning and treatment attempts, including unnecessary radionuclide injections, which ideally should be avoided to minimize patient dose and maintain maximum efficiency in the clinic. The ability to predict BgRT treatment feasibility based on initial diagnostic PET parameters would significantly improve patient selection and treatment planning efficiency. Our goal was to establish size-specific SUVmax thresholds for small targets that would reliably predict successful multi-target BgRT deliveries, providing practical guidance for clinical implementation.

## 2. Materials and Methods

### 2.1. Phantom Design

**Figure 1:**
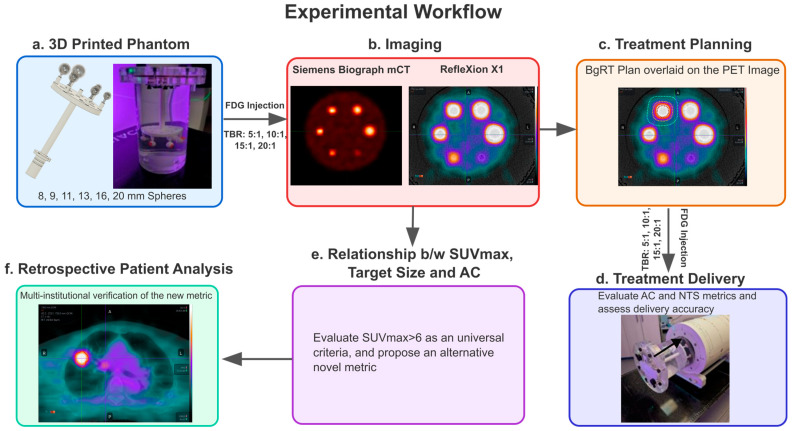
The experimental workflow followed in this study: (**a**) depicts a 3D model of the phantom containing six spherical targets ranging from 8 to 20 mm in diameter on the left, and the phantom housed inside a cylindrical insert on the right. (**b**) The spheres and the background cylinder were injected with varying amounts of FDG to achieve different target-to-background ratios (TBRs) (5:1, 10:1, 15:1, 20:1) to achieve different SUVmax values. Imaging was then performed on the Siemens Biograph mCT for SUVmax quantification and functional modeling on the Reflexion to quantify AC and NTS. (**c**) shows the treatment plan for 13 mm sphere overlaid on the PET image. A total of 24 plans were attempted to be optimized. (**d**) The targets were injected with FDG again, with the same TBR. The complete assembly integrated with ArcCHECK for delivery verification is shown. The arrow indicates that for the actual experiments, the cylinder containing the phantom was pushed all the way inside the cavity. (**e**) Correlation analysis was performed between SUVmax, AC and NTS. (**f**) clinical data from four institutions was used to validate this phantom-derived metric.

A custom 3D-printed phantom containing six spherical targets of 8 mm, 9 mm, 11 mm, 13 mm, 16 mm, and 20 mm diameter was developed in Fusion 360 ^®^ (Autodesk, San Rafael, CA, USA). BioMed Clear Resin was used on a Formlabs (Somerville, MA, USA) 3D printer to print the 3D phantom. The phantom was then integrated within a cylindrical insert compatible with the ArcCHECK (Sun Nuclear Corporation, Melbourne, FL, USA), enabling both imaging and subsequent delivery verification. The 3D model of the phantom is shown in Figure 1a, along with the phantom housed inside the cylinder, which was then inserted into the cylindrical cavity in the ArcCheck (Figure 1d). Figure 1b shows an example PET image taken on the Siemens Biograph mCT and the Reflexion X1 system, after the targets were injected with FDG-F18. To minimize leakage, we employed O-rings at all junctions where components were joined. This ensured a tight seal and helped prevent any unintended leaks during operation.

### 2.2. Target Preparation, Image Acquisition and Evaluating Relationship Between AC, NTS, SUVmax and Target Size

To systematically investigate the relationship between target size, SUVmax, and resulting AC values, we conducted experiments with varying target-to-background ratios (TBRs). Targets and the background were injected with 18F-fluorodeoxyglucose (18F-FDG) with different activities depending on the desired target-to-background ratio (TBR). Four different TBRs (5:1, 10:1, 15:1, and 20:1) were used while maintaining a consistent background activity of 5 kBq/mL across all experiments. This approach allowed us to achieve a range of SUVmax values for each target size, simulating various levels of PET avidity encountered in clinical practice.

To ensure homogeneity, the targets included two access points to facilitate air bubble removal, which were actively purged during preparation. Homogeneity was further confirmed by inspecting the CT images for the absence of air pockets.

After preparation, the phantom underwent imaging on two systems. First, diagnostic PET-CT was performed on the Siemens Biograph mCT (Siemens Healthineers, Erlangen, Germany) for SUVmax quantification, representing the initial patient evaluation typically used in clinical practice. The PET acquisition consisted of 3 min per bed position (180 s), with images reconstructed using point spread function (PSF) with time-of-flight (TOF) correction (2 iterations, 21 subsets) and a 3D Gaussian post-reconstruction filter (2.0 mm FWHM). The reconstruction matrix was 200 × 200 with a pixel size of 4.07 mm and slice thickness of 3 mm. Attenuation correction was performed using low-dose CT data, with images corrected for decay, scatter, random coincidences, normalization, and dead time. Subsequently, functional modeling was performed on the RefleXion X1 for BgRT planning and AC quantification. The relationship between AC, SUVmax, and its dependence on target size was then analyzed using data from both imaging systems.

MIM v 7.3.3 (MIM Software Inc., Cleveland, OH, USA) was used to contour the PET-avid lesions for phantom study. Target volume delineation was performed using PET Edge^®^+ within the MIM Software environment (MIM Software Inc., Cleveland, OH, USA). This gradient-based segmentation algorithm identifies tumor boundaries by detecting the steepest gradient in PET signal intensity, providing more consistent and reproducible contours compared to threshold-based methods. Previous validation studies have demonstrated that gradient-based techniques offer superior accuracy for contouring PET-avid lesions, particularly for smaller spherical targets with diameters < 20 mm [10]. It is important to emphasize that the volumes defined in this phantom study strictly represent the PET-avid regions as determined by the gradient segmentation algorithm, rather than clinical gross tumor volumes (GTVs) that might incorporate additional anatomical or clinical information specific to different disease sites. To ensure methodological consistency and enable fair comparison of volume metrics across all targets, all volumetric measurements were performed exclusively within the MIM software environment for the phantom study.

### 2.3. Treatment Planning

BgRT treatment plans were generated using the RefleXion SCINTIX treatment planning system. As indicated above, PTVs were created by adding a 5 mm margin to each of the six GTVs investigated, and a 10 mm margin was added to GTVs to create BTZs. A standard fractionation scheme of 10 Gy per fraction for 5 fractions was prescribed to cover the PTV (i.e., each of the spheres). A total of 24 plans were optimized: 6 Target sizes × 4 Target-to-background ratios. However, the X1 system’s built-in safety features prevented delivery when AC values fall below 5 kBq/mL, as targets with insufficient contrast cannot be reliably tracked during treatment delivery.

### 2.4. Treatment Delivery and Validation

The phantoms were filled with FDG again for treatment delivery, and for all cases where AC exceeded 5 kBq/mL, BgRT plans were delivered on the RefleXion X1 system. Delivery accuracy was validated using ArcCHECK with gamma criteria of 3%/2 mm and 3%/3 mm (dose difference/distance to agreement), 10% dose threshold and global normalization, and the treatment planning system dose distribution was used as the reference plan [11]. The plans were delivered sequentially starting from the smallest targets. These measurements provided quantitative assessment of the delivery accuracy for targets of different sizes and uptake levels.

### 2.5. Evaluation of Retrospective Analysis of Patient Data

To validate the phantom-derived findings in a clinical context, a retrospective analysis was conducted of 18 patients from 4 different institutions with small lesions (<7.5 cc) who had undergone BgRT planning using the RefleXion X1 system. This upper volume threshold represents the size range where both metabolic activity and target volume contribute proportionally to BgRT eligibility. Additionally, patient selection was restricted to those who had undergone imaging within 75 min post-injection, as longer delays would result in artificially lower Activity Concentration (AC) values. The analyzed lesions varied in size (1.2 cc to 7.4 cc), location (lung and bone), and metabolic activity (SUVmax range: 3.6–37.3). For each patient, SUVmax and PET-avid volume measurements were extracted from diagnostic PET-CT scans, along with corresponding AC and Normalized Target Signal (NTS) values obtained during BgRT modeling studies. To minimize variability from inconsistent contouring practices across institutions, specific instructions were provided for volume measurements. Institutions were requested to report PET-avid tumor volumes specifically, rather than anatomically defined GTVs that might include non-avid regions. When GTVs contained both PET-avid and non-avid components, institutions were asked to provide measurements of the PET-avid volume separately. This clinical dataset allowed for comparison of the relationship between target volume, SUVmax, and AC values with the patterns observed in the controlled phantom study, providing validation in a clinical context. The current clinical criterion (SUVmax ≥ 6) was evaluated for its efficacy in predicting successful BgRT planning in this patient cohort. Finding limitations in this approach, particularly for smaller targets, a more robust predictive metric was sought that would account for both uptake and target size. To rigorously validate any proposed alternative criterion, non-parametric bootstrap analysis [12,13,14] was performed with 1000 iterations to account for statistical uncertainty and establish confidence intervals. This retrospective analysis utilized fully anonymized patient data from a collaborative BgRT treatment registry in compliance with institutional review board requirements.

## 3. Results

### 3.1. Relationship Between SUVmax, AC, NTS and Target Size

**Figure 2:**
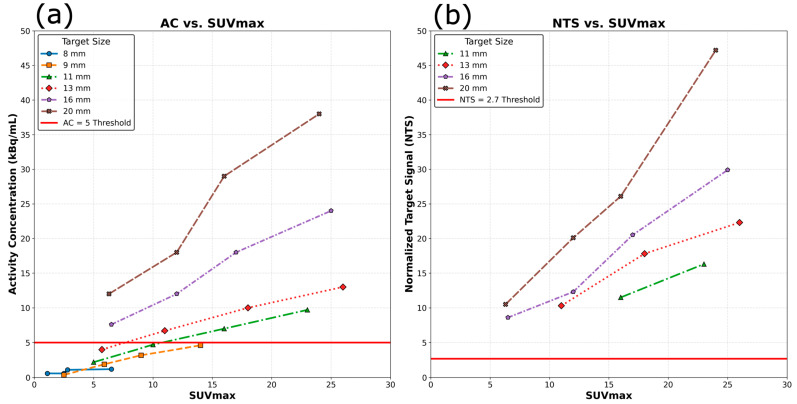
Relationship between SUVmax and AC and NTS for targets of different sizes. (**a**) shows the activity Concentration (AC) versus SUVmax for different target sizes. The critical AC > 5 kBq/mL threshold required for successful BgRT planning is shown in red. Smaller targets require disproportionately higher SUVmax values to achieve sufficient AC. (**b**) depicts the Normalized Target Signal (NTS) versus SUVmax demonstrating a linear relationship across all target sizes. The NTS threshold is shown in red (NTS = 2.7). The NTS is dependent on the AC, and if AC < 5 kBq/mL is detected, the NTS is not calculated by the RefleXion system.

The built-in safety metrics allowed the SCINTIX system to successfully plan treatments for 13 of 24 BgRT experiments, all of which achieved AC > 5 kBq/mL. The AC and NTS for different target sizes and their relationship with SUVmax is shown in Figure 2. A linear relationship between SUV max and NTS was observed for all target sizes studied. For the largest targets (16 mm and 20 mm), SUVmax > 6 consistently yielded AC > 5 kBq/mL, enabling successful BgRT planning and delivery across all tested target sizes. This finding validates the current clinical criterion of SUVmax ≥ 6 for targets of this size range. Medium-sized targets (13 and 11 mm) demonstrated a more nuanced relationship. While SUVmax > 6 was achieved under most conditions, the AC > 5 kBq/mL criterion was only met when SUVmax exceeded 10 for the 13 mm target and 15 for the 11 mm target, indicating that the current SUVmax ≥ 6 criterion is inadequate for targets of these sizes. The smallest targets tested (8 mm and 9 mm) revealed a critical limitation. These targets could not meet the AC > 5 kBq/mL threshold despite achieving SUVmax values up to 14, suggesting that targets <10 mm may be fundamentally challenging for BgRT under current technical constraints. The NTS is dependent on the AC, and if AC < 5 kBq/mL is detected, the NTS is not calculated by the system, which is why Figure 2b only shows data for successful plans.

### 3.2. Treatment Delivery Accuracy

**Figure 3:**
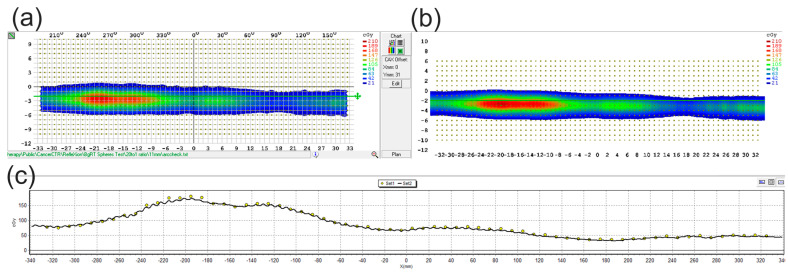
Treatment delivery verification. (**a**,**b**) ArcCheck measured distribution for the 11 mm target shown on the left, with the reference TPS distribution on the right for the 20:1 target-to-background ratio. The gamma passing rate for this delivery was 91.6% for 3%/2 mm and 97.7% for 3%/3 mm. Line profile across the green horizontal line in (**a**,**b**) is shown in (**c**).

Figure 3 shows an ArcCheck measured distribution and the planned dose for the 11 mm target (TBR: 20:1). All successful BgRT deliveries maintained clinically acceptable accuracy (3%/3 mm), with a mean gamma passing rate of 92.4% ± 5.0% (range: 81.9% to 100%) for 3%/2 mm criteria and improved to a mean pass rate of 97.6% ± 1.9% (range: 93.8% to 100%) using 3%/3 mm criteria as shown in Table 1. Detailed analysis of gamma passing rates showed no significant correlation between target size, SUVmax and delivery accuracy suggesting that the AC > 5 kBq/mL threshold effectively ensures adequate delivery quality regardless of target volume when this threshold is met. The current iteration of the RefleXion X1 linac can only treat multiple targets sequentially; therefore one reason for this lack of correlation can be partially attributed to the sequential experimental design, where smaller targets were treated first. It should be noted that AC and NTS for each target are evaluated again at treatment to ensure sufficient signal quality at the time of delivery. With each delivery requiring approximately 20–30 min, the F-18 radiotracer underwent significant decay by the time larger targets were treated. This progressive decay counterbalanced the initially higher SUVmax, AC, and NTS values for larger targets, effectively normalizing the signal strength across the treatment sequence. Planning and treatment AC and NTS values are presented in Table 2 for each target size and target-to-background ratio (TBR). The 8 and 9 mm targets are omitted as they universally failed to meet the AC threshold. The sequential treatment approach meant that the smaller targets treated early in the sequence demonstrated minimal differences between planning and treatment values, while the largest targets (20 mm) exhibited substantial disparities, potentially contributing to their lower-than-expected gamma passing rates.

### 3.3. Volumetric-SUVmax Product as a Novel Predictive Metric

**Figure 4:**
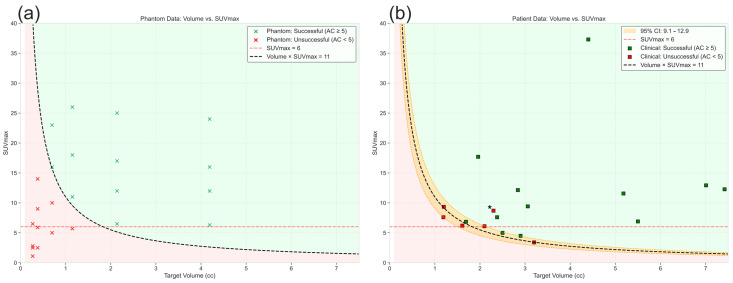
Volume × SUVmax product as a predictive metric for successful BgRT planning. (**a**) Shows the data from the phantom study where the data points represent experimental conditions categorized by planning success (green) or failure (red). The proposed threshold of Volume × SUVmax > 11 (dashed line) accurately predicts planning success across all target sizes for the phantom study. (**b**) shows the patient data analysis (N = 18). The 95% confidence interval was established using bootstrapping (number of bootstrap samples = 1000). The phantom derived threshold (11) predicted treatment eligibility in 15 out of the 18 clinical cases. The red dotted line represents the current SUVmax threshold.

Our findings above demonstrate that the SUVmax threshold required for successful BgRT planning is significantly dependent on target size or volume, with an inverse relationship between these parameters as shown in Section 3.1. This observation led us to investigate whether diagnostic PET-CT data could be used to prospectively identify suitable BgRT candidates prior to treatment planning. Analysis of successful plans revealed a consistent relationship between target volume, SUVmax, and planning success. Product of PET-avid target volume (cc) and SUVmax greater than 11 consistently predicted successful BgRT planning across all target sizes used in the phantom study. This criterion accurately classified all 24 experimental conditions in our phantom study, as illustrated in Figure 4a, which plots the SUVmax × Volume relationship against our experimental data points. While a diameter-based metric can be derived (as discussed later in the discussion section), volume was selected for the predictive model for several reasons. First, PET signal generation and AC calculation fundamentally depend on radiotracer accumulation throughout the entire three-dimensional tumor volume, not just linear dimensions. Second, although our phantom study utilized spherical targets for controlled evaluation, clinical lesions can be irregular in shape. For such non-spherical targets, volume measurement captures the true extent of radiotracer distribution more accurately than any single linear dimension.

As mentioned earlier, to validate this finding in clinical scenarios, a retrospective analysis focusing on patients with smaller targets (<7.5 cc) was conducted. Among the 18 patients analyzed, the Volume × SUVmax > 11 criterion based on the phantom study correctly predicted treatment eligibility in 15 cases. To assess the statistical reliability of our Volume × SUVmax > 11 threshold, a bootstrap analysis with 1000 resamples of patient dataset was performed. For each bootstrap sample, 100 candidate thresholds between 4 and 35 were evaluated by calculating the classification accuracy (proportion of correct predictions) when using that threshold to predict treatment success. The range of 4–35 was selected to encompass all observed product values in our phantom dataset with sufficient margin. The threshold that maximized classification accuracy for each bootstrap sample was selected, resulting in a distribution of 1000 optimal threshold values. When multiple thresholds achieved identical maximum classification accuracy within a bootstrap sample, the lowest threshold was used which is consistent with the goal of optimizing clinical utility. It should be noted here that while we selected the lowest threshold among equally performing options to maximize clinical utility, a random selection approach may provide a more conservative estimate of threshold uncertainty. This approach yielded a 95% confidence interval (CI) of 9.1 to 12.9 with a mean of 11.3 for the optimal threshold value as shown in Figure 4b. The bootstrap threshold histogram for patient data is shown in the supplementary section in Figure S1. The narrow width of this confidence interval supports the robustness of the proposed criterion. As visualized in Figure 4b, the uncertainty band (orange shaded region) encompasses the transition zone between successful and unsuccessful BgRT planning outcomes, with minimal misclassifications. One notable outlier case with a product value of 20 (marked with asterisk in Figure 4b) failed to meet the AC threshold, contrary to the expected pattern.

Further investigation revealed that for this patient, there was high uptake in the background shell used in AC calculation which artificially reduced the measured AC. Nonetheless, the AC was 4.6, which made it a borderline failure. Note that the CI were established by removing the outlier. Excluding the outlier, the threshold derived from the phantom dataset (i.e., 11) yielded an accuracy of 88%, when applied to the patient dataset. Furthermore, the two failures are within the 95% CI.

**Figure 5:**
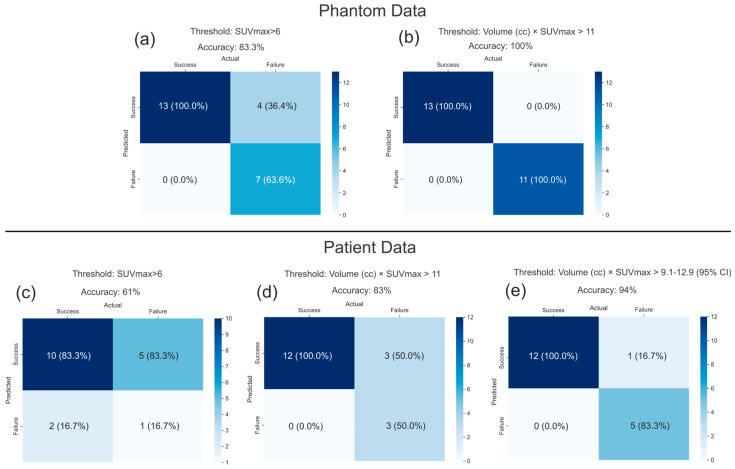
Error/Confusion matrices comparing PET-based detection criteria for both phantom and patient data. The matrices illustrate the predictive performance of two detection criteria: the current SUVmax > 6 threshold and the new proposed Volume × SUVmax > 11 threshold for both the phantom and the patient dataset. In (**a**), error matrix for the phantom dataset using the current recommended threshold is shown. An accuracy of 83.3% was achieved, however using the new volume-scaled SUVmax metric proposed in this study, the accuracy increased to 100% (**b**), (**c**–**e**) show the error matrices for the patient dataset. Using the current recommendation of SUVmax > 6, an accuracy of 61% was achieved. However, this increased to 83% when using the new proposed threshold (**d**). (**e**) shows the error matrix if all points falling within 95% CI were to be counted as correctly classified. In that case, the classifying accuracy increases to 94%.

The predictive performance of the current criterion versus the volume-scaled SUVmax criteria proposed in this study is shown in Figure 5. Figure 5a shows the error matrices for the phantom dataset; using the current recommendation of SUVmax > 6, an accuracy of 83.3%, sensitivity of 100% and specificity of 63.6% were obtained. However, as mentioned previously, the volume scaled SUVmax metric presented here achieved an accuracy of 100% as shown in Figure 5b. Figure 5c shows the multi-institutional patient data error matrix with the traditional SUVmax ≥ 6 criterion. This yielded an accuracy of 61%, sensitivity of 83.3% and specificity of 16.7%. Figure 5d shows improved performance (83.3% accuracy, 100% sensitivity and 50% specificity) when applying the Volume × SUVmax > 11 threshold. Figure 5e demonstrates further improvement to 94% accuracy, 100% sensitivity and 83.3% specificity, when accounting for measurement uncertainty in the Volume × SUVmax > 11 criterion.

## 4. Discussion

This study demonstrates that the relationship between target size and SUVmax significantly impacts the feasibility of Biology-guided Radiotherapy (BgRT) on the RefleXion platform for small targets. While the current SUVmax ≥ 6 recommendation proves adequate for targets ≥ 16 mm, smaller targets require substantially higher SUVmax values to achieve sufficient signal for BgRT planning and delivery. Analysis of different volumes and uptake scenarios across 24 phantom configurations led to the development of a novel predictive criterion: Volume (cc) × SUVmax > 11, which more accurately predicts treatment success for small targets than SUVmax-only criteria. This composite metric has strong physical basis. The total number of detectable positron annihilation events is proportional to both radiotracer concentration (reflected by SUVmax) and the volume of tissue containing that tracer. Mathematically, total detectable PET signal ∝ Concentration × Volume, which forms the physical basis for our metric. For BgRT delivery, the RefleXion system must detect sufficient total PET signal to distinguish tumor from background and enable real-time tracking which depends inherently on both metabolic intensity and spatial extent.

This metric serves particularly well for initial screening of patients for BgRT, potentially reducing unsuccessful planning attempts and associated costs including unnecessary radionuclide injections. However, it is important to recognize optimal application domain of this metric. This metric is valuable for smaller targets (<~7 cc), where the relationship between size and required SUVmax is most critical. Small lesions are affected by partial volume effects, where limited spatial resolution causes signal averaging with surrounding background tissue. When lesion dimensions approach twice the scanner’s spatial resolution (typically 4–5 mm FWHM for modern PET systems), the measured SUVmax becomes artificially reduced, often by 20–50% compared to true values. In our study, the Siemens Biograph mCT has a pixel spacing of 4.07 mm and slice thickness of 3 mm, meaning targets below 10 mm diameter experience substantial partial volume effects. This explains why 8–9 mm phantom targets failed to achieve AC > 5 kBq/mL even at measured SUVmax values of 14. The true metabolic activity was likely much higher but could not be fully captured due to spatial resolution limitations. The Volume × SUVmax product compensates for these competing effects by capturing total metabolic burden rather than peak concentration alone. For larger tumors, the volume factor begins to dominate the product, potentially overestimating eligibility for tumors with relatively low metabolic activity but substantial size.

While tumor diameter was used throughout the initial analysis (i.e., for the phantom study), the predictive metric was developed using volume to better capture the three-dimensional nature of PET signal generation. This approach is expected to produce superior predictive accuracy compared to diameter-based metrics, especially for irregularly shaped targets. For clinical implementation with approximately spherical lesions, the Volume × SUVmax > 11 criterion can be translated to a diameter-based formula using the sphere volume equation V = (4/3) πr^3^ = (π/6) d^3^, where d is the maximum diameter in centimeters. This yields a simplified criterion of d^3^ × SUVmax > 21 for rapid clinical assessment using maximum diameter measurements. This metric is particularly valuable as both target diameter and SUVmax are routinely reported in diagnostic PET-CT reports, allowing clinicians to quickly assess BgRT eligibility during initial consultation without requiring specialized software or analysis.

Our study also provides valuable insight into the optimal treatment sequence for multi-target treatments on the RefleXion system. Intuitively, it is expected that smaller targets should, in general, be prioritized over larger targets. This is evident in the data presented in Table 2, where the difference between planning and treatment AC for the larger targets was as large as 50%, as they were treated at the end. Although this holds true for the systematic phantom study performed here, certain clinical scenarios might warrant alternative approaches. To translate the above findings into clinical guidance for sequential multi-target treatment on the RefleXion system, we calculated the maximum treatment window which was defined as the time from radiotracer injection until AC decreases below 5 kBq/mL, for various target size and SUVmax combinations (Table 3). These treatment windows were calculated using the data presented in Figure 2 and exponential decay of radioactivity based on F-18 half-life. These calculations reveal that treatment window duration is determined by both target size and uptake intensity, with important implications for treatment sequencing. Consider a case with a ~2 cc target with SUVmax 5–7 alongside a smaller ~0.7 cc target with SUVmax > 20. Contrary to the approach taken in the systematic phantom study performed here, the larger target should be prioritized due to its shorter treatment window (1.5 h versus 2.5 h), highlighting how the interplay between size and uptake ultimately determines optimal treatment sequence.

The retrospective patient analysis revealed a threshold which was similar to the one derived from the phantom study. While this metric correctly classified all 24 phantom data points, patient data showed greater variability, reflected in the established confidence intervals (9.1–12.9). This variability primarily stems from inconsistent contouring practices across institutions. In our controlled study, we minimized this effect by consistently using MIM’s PET Edge+ tool for automated segmentation. For external institutions, we requested both total GTV measurements and specific PET-avid volumes when GTVs contained non-avid regions. It should also be noted that tumors typically have heterogenous FDG uptake, which also contributes to the increased uncertainty.

Alternative PET-based metrics were also considered in our analysis. Total Lesion Glycolysis (TLG) [15,16], calculated as SUVmean × tumor volume, is a widely used parameter in PET imaging that quantifies the total metabolic activity within a lesion. While TLG offers a comprehensive assessment of tumor burden, it introduces significant variability in the BgRT planning context due to its dependence on SUVmean. Unlike SUVmax, which represents a single highest-value voxel, SUVmean is highly sensitive to contouring decisions and threshold selections, making it inherently more subjective and operator-dependent. In nuclear medicine/radiology reporting, SUVmean is often calculated by placing a sphere around the tumor, and therefore it is highly correlated with the sphere size. Nonetheless, we did observe that an SUVmean > 5, in general, successfully predicted BgRT eligibility. The RefleXion system itself uses SUVmax for eligibility screening rather than SUVmean-based metrics, recognizing the greater reproducibility and lower variability of maximum uptake values. The Volume × SUVmax approach maintains this advantage while accounting for the critical size dependency that was observed. Furthermore, this criterion can be easily calculated from standard diagnostic PET-CT data to better manage the eligibility of patients for BgRT treatments.

It should be noted that similar phantom-to-clinical validation approaches have been employed for partial volume effect correction [17,18], and determining optimal PET window levels for radiation therapy target delineation [10]. While the phantom methodology itself follows standard practices, we wish to emphasize the novelty of our specific contribution. This is the first size-dependent eligibility criterion for BgRT, as previous eligibility relied solely on SUVmax ≥ 6 without accounting for the critical size dependency we have demonstrated. Our work demonstrates that a composite Volume × SUVmax metric outperforms SUVmax-only criteria for small targets through systematic characterization that controlled variation in both target size (6 sizes) and uptake (4 target-to-background levels) across 24 configurations. This is an experiment which would be impossible to replicate systematically in clinical settings.

It is crucial to emphasize that the Volume × SUVmax > 11 threshold established in this study should not be considered a constant, but rather a baseline metric that will evolve with technological advancements. The current threshold reflects the capabilities of the first-generation RefleXion X1 system with its specific PET detector configuration and reconstruction algorithms. As hardware and software improvements emerge in subsequent iterations of BgRT platforms, it is expected that this threshold will likely decrease, enabling successful treatment of progressively smaller and less PET-avid lesions. Enhanced PET detector sensitivity, improved spatial resolution, more sophisticated reconstruction algorithms, and advanced noise reduction techniques will collectively contribute to better signal detection and lead to a more universal, size-independent relationship between SUVmax and AC. Nonetheless, institutions adopting BgRT technology should follow the framework presented here to establish the criterion to their specific hardware configuration.

A significant limitation of this study is the absence of respiratory or physiological motion in the experimental setup. While the phantom experiments provided a controlled environment to establish the fundamental relationship between target size, SUVmax, and successful BgRT planning, they did not account for the complex dynamics of tumor motion in clinical scenarios. Respiratory motion can substantially impact both PET image quality and treatment delivery accuracy [9]. Motion can lead to signal blurring, effectively reducing the apparent SUVmax and Activity Concentration, potentially requiring higher initial SUVmax values than the static model suggests. For targets in highly mobile regions such as the lower lungs or upper abdomen, the Volume × SUVmax threshold may need adjustment to compensate for motion-induced signal degradation. Future studies incorporating dynamic phantoms with programmed respiratory patterns are required as they will offer valuable insights into how different motion amplitudes and frequencies affect the minimum SUVmax.

## 5. Conclusions

Target size significantly influences BgRT treatment eligibility, with a non-linear relationship between target size and required SUVmax. Our comprehensive phantom study demonstrates that while the current clinical criterion of SUVmax ≥ 6 is sufficient for larger targets (≥16 mm), smaller lesions require substantially higher SUVmax values to achieve the minimum Activity Concentration threshold of 5 kBq/mL necessary for successful BgRT planning and delivery. We propose a new simple criterion, Volume × SUVmax > 11 (9.1–12.9–95% CI), that more accurately predicts treatment eligibility for small targets (<7 cc) without requiring prior knowledge of AC values. This metric provides clinicians with a practical tool for patient selection using standard diagnostic PET-CT data. Our findings also established practical treatment window guidelines based on target size and uptake characteristics, enabling optimized scheduling and sequencing of multi-target BgRT delivery.

## Figures and Tables

**Table 1:** Gamma analysis passing rates (%) for different sphere sizes and target-to-background ratios (TBR). Values show 3%/2 mm criteria with 3%/3 mm in parentheses. Spheres of 8–9 mm diameter were not deliverable for TBR or target size. X indicates configurations where treatment delivery was not feasible.

TBR	11 mm	13 mm	16 mm	20 mm
5:1	X	X	89.7 (93.8)	91.7 (96.0)
10:1	X	86.0 (96.4)	99.2 (100)	94.1 (97.6)
15:1	93.4 (99.7)	100.0 (100.0)	91.9 (98.0)	89.9 (96.1)
20:1	91.6 (97.7)	81.9 (95.7)	97.0 (100.0)	94.4 (97.6)

**Table 2:** AC (kBq/mL) and NTS values for different sphere sizes during planning and treatment (treatment values in parentheses). During each injection session, smaller targets were delivered first (longer detection thresholds), resulting in lower AC values for larger targets during treatment due to F18 decay. Spheres of 8–9 mm diameter were not deliverable at any tested Target-to-background ratio (TBR). X indicates configurations where treatment delivery was not feasible.

TBR	11 mm		13 mm		16 mm		20 mm	
	AC (kBq/mL)	NTS	AC (kBq/mL)	NTS	AC (kBq/mL)	NTS	AC (kBq/mL)	NTS
5:1	X	X	X	X	7.6 (7.7)	8.6 (6.3)	11.7 (9.7)	10.5 (9.7)
10:1	X	X	6.7 (6.7)	10.3 (7.0)	11.9 (8.9)	12.3 (11.3)	18.4 (9.2)	20.1 (15.3)
15:1	7.0 (8.3)	11.5 (9.7)	10.4 (8.6)	17.8 (9.8)	17.8 (10.8)	20.5 (15.5)	29.2 (9.9)	26.1 (16.1)
20:1	9.7 (11.3)	16.3 (14.7)	13.3 (11.3)	22.3 (14.7)	23.9 (11.8)	29.9 (20.3)	37.8 (12.9)	47.2 (23.7)

**Table 3:** Maximum Treatment Window Before AC < 5 kBq/mL.

SUVmax	Target Size			
	11 mm (0.70 cc)	13 mm (1.15 cc)	16 mm (2.14 cc)	20 mm (4.19 cc)
5–7	N/A	N/A	1.5 h	2.5 h
8–12	N/A	1 h	2.5 h	3.5 h
13–17	0.5 h	2 h	3.5 h	4.5 h
18–22	1.5 h	3 h	4.5 h	5.5 h
23–27	2.5 h	4 h	5.5 h	6.5 h
≥28	3.5 h	5 h	6.5 h	7.5 h

Based on F-18 half-life (109.8 min) and target AC threshold of 5 kBq/mL.

## Data Availability

Research data are stored in an institutional repository and will be shared upon request to the corresponding author.

## Supplementary Materials

The following supporting information can be downloaded at: https://www.mdpi.com/article/10.3390/cancers17223645/s1, Figure S1: Threshold Value.
