# Supplementary material for: Multi-Institutional Verification of a Novel Predictor (Volume-Scaled SUVmax) for Successful Biology-Guided Radiotherapy Delivery of Small Targets
Source: Cancers (Basel). 2025 Nov 13;17(22):3645. doi: 10.3390/cancers17223645 (PMC12651831; doi:10.3390/cancers17223645)
Supplement: Supplementary file 1 [file cancers-17-03645-s001.zip › cancers-3931416-supplementary.pdf]

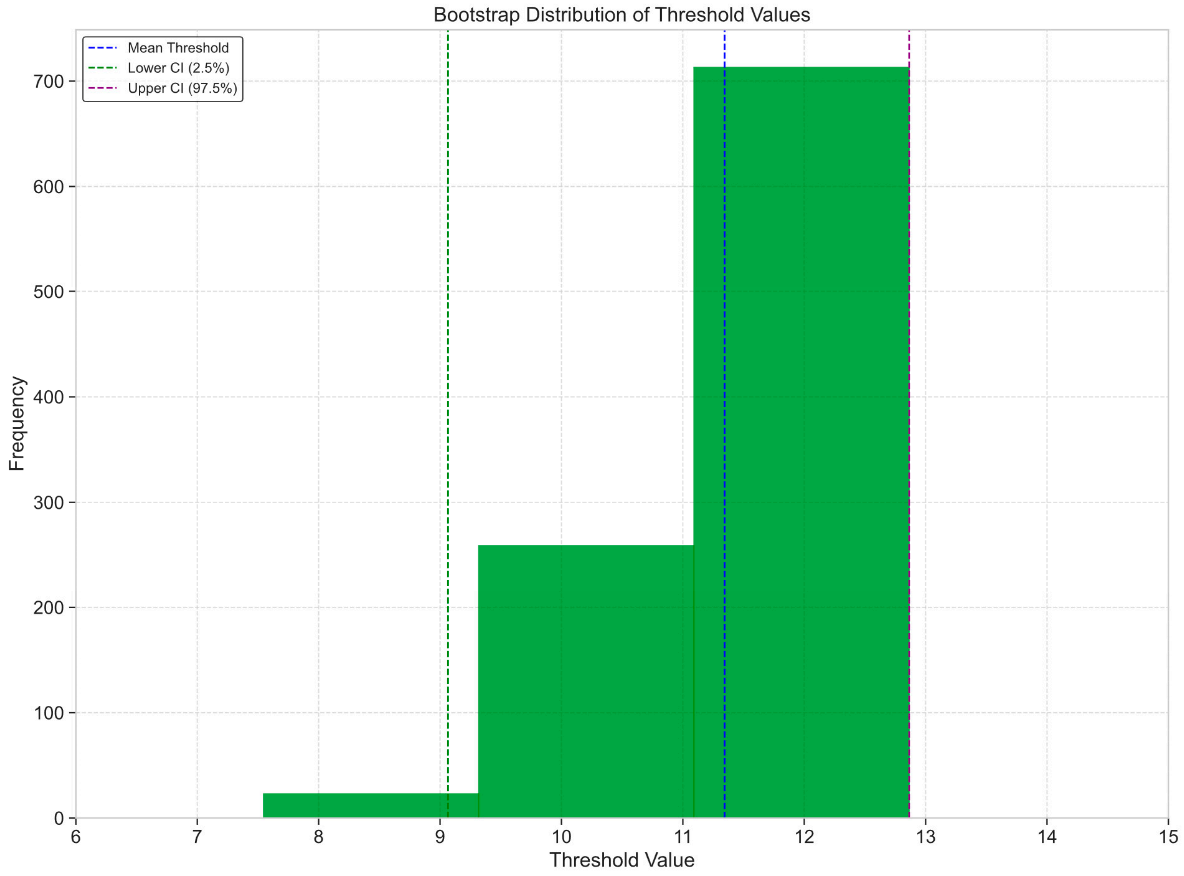

Figure S1: Distribution of optimal Volume × SUVmax threshold values derived from 1,000 bootstrap resamples of clinical data. The mean and 95% confidence intervals are also plotted.
